# Comparison of the effects of different positive end-expiratory pressure levels on respiratory mechanics and oxygenation in laparoscopic surgery: A protocol for systematic review and network meta-analyses

**DOI:** 10.1097/MD.0000000000013396

**Published:** 2018-11-30

**Authors:** Min Wang, Nan Geng, Ya Gao, Yan Zhang, Yingbin Wang, Xiping Shen, Jinhui Tian, Bo Wang

**Affiliations:** a Department of Anesthesiology, Second Hospital of Lanzhou University; b The Second Clinical Medical College of Lanzhou University; c Evidence-Based Medicine Center, School of Basic Medical Sciences; d Institute of Epidemiology and Health Statistics, School of Public Health, Lanzhou University; e Department of Nursing, Rehabilitation Center Hospital of Gansu Province, Lanzhou, China.

**Keywords:** laparoscopic surgery, network meta-analysis, oxygenation, positive end-expiratory pressure, respiratory mechanics, systematic review

## Abstract

**Background:**

Several studies have observed the good effects of positive end-expiratory pressure (PEEP) application in laparoscopic surgeries, such as counteracted intraoperative atelectasis, improved respiratory mechanics, and gas exchange. However, evidence of systematic comparisons of different PEEP levels is short, and the optimal level of PEEP during laparoscopy remains unknown and controversial. The study aims to compare the effects of different PEEP levels on respiratory mechanics and oxygenation in laparoscopic surgery using network meta-analyses.

**Methods:**

To identify relevant studies, a systematic search will be conducted among electronic databases, including PubMed, Cochrane Library, EMBASE, and Web of Science. We will include randomized controlled trials (RCTs). The risk of bias in the included RCTs will be assessed using the Cochrane bias risk tool. Network meta-analysis will be performed using STATA 15.0, and R 3.4.1 software.

**Results:**

This study is ongoing and the results will be published in a peer-reviewed journal.

**Conclusion:**

The results of this study will be sent to clinicians and healthcare providers in the National Health Service, which is expected to help clinicians make more informed treatment decisions and facilitate further research on the use of PEEP during surgery.

**PROSPERO registration number:**

CRD42018093537.

## 1 Introduction

In recent years, laparoscopic surgeries have been replacing many laparotomy procedures due to its advantages of the shorter length of hospital stay, minimal postoperative pain, a reduction in inflammatory and metabolic responses, and quick recovery.^[1–3]^ However, laparoscopic surgery may raise concerns about the adverse effects associated with increased intra-abdominal pressure due to the induction of pneumoperitoneum, which leads to cranial shift of the diaphragm, resulting in the compression of lung tissue in basal lung regions, decreased both chest wall compliance and functional residual capacity (FRC) and may result in intraoperative atelectasis and impair gas exchange.^[4–8]^ General anesthesia additionally causes impairment in pulmonary gas exchange, and decreases blood oxygenation by development of atelectasis in dependent lung regions, with an increase in pulmonary shunt leading to impairment of oxygenation associated with reduction of FRC, which dues to decreased inspiratory muscle tone, increased abdominal pressure and altered thoracic volume.^[8–14]^ Also, trendelenburg position influences the abdominal pressure through gravity, causes a decrease in lung compliance and an increase in airway resistance (RAW), thus may result in additional loss of FRC and further changes in the respiratory system.^[15,16]^ Perioperative atelectasis is generally accepted to affect gas exchange and as a major cause for development of postoperative hypoxia.^[17]^ Positive end-expiratory pressure (PEEP) is defined as the application of positive pressure to the airway at the end of expiration. PEEP improves pulmonary oxygen exchange through prevention of the collapse of airways, the redistribution of pulmonary blood flow, increased FRC, also improves pulmonary compliance and ventilation perfusion abnormalities.^[7,18,19]^ It was reported that the application of PEEP plays an important role in counteracting intraoperative atelectasis, improved respiratory mechanics and ventilation-perfusion abnormalities during laparoscopic surgeries.^[9,20–22]^ Besides, previous studies showed that PEEP applications provide positive effects on respiration parameters in patients undergoing laparoscopic cholecystectomy.^[20,23]^ However, evidence of systematic comparisons of different PEEP levels are short, and the optimal level of PEEP during laparoscopy remains unknown and controversial.^[24]^ Network meta-analysis (NMA) has been considered to extend conventional meta-analyses on multiple treatments for a given condition.^[25]^ It becomes increasingly popular since allowing for estimation of the relative effectiveness among all interventions and rank ordering of the interventions even if head-to-head comparisons are lacking.^[26]^ In this study, we will collect all relevant evidence to investigate the effects of intraoperative PEEP application during pneumoperitoneum on respiratory mechanics and oxygenation through an NMA, in order to provide evidence for clinical practice and further research.

## 2 Methods

### 2.1 Design and registration

We registered on the international prospective register of systematic review (PROSPERO) to publish our study protocol. The procedures of NMA will be conducted according to the PRISMA extension statement for reporting of systematic reviews incorporating network meta-analyses of healthcare interventions.^[27]^

### 2.2 Eligibility criteria

#### 2.2.1 Type of studies

Any randomized controlled trial (RCT) regardless of sample size will be included if met the following criteria: include adult patient undergoing laparoscopic surgery; compare any administration of PEEP during laparoscopic surgery, including no PEEP and multiple PEEP levels; and the trials should contain at least one of the parameters of respiratory mechanics and oxygenation. Studies will be excluded if there are insufficient data to summarize the results after trying to contact the author about data provision and duplicate publications.

#### 2.2.2 Patients

We will include studies, which contain adult patients who received PEEP during laparoscopic surgeries. Adolescents (under 18 years of age) and patients with cerebrovascular, respiratory, cardiovascular, and metabolic disease will be excluded from the present study. We will put no limitations on age, gender, and nations.

#### 2.2.3 Interventions and comparators

PEEP is defined as the application of positive pressure to the airway at the end of expiration. The interventions are the application of PEEP during laparoscopic surgery, including no PEEP and multiple PEEP levels such as 5, 10, and 15 cm H_2_O. And any level of PEEP compared with each other will be included, such as ZEEP (group without PEEP) or another level of PEEP.

#### 2.2.4 Outcome of interest

The primary outcomes of interest are the respiratory mechanics’ parameters, such as peak airway pressure, mean airway pressure, plateau pressure (P plateau), RAW, and respiratory compliance. The secondary outcome is the parameter of oxygenation.

### 2.3 Search strategy

A systematic search will be performed using PubMed, EMBASE, Cochrane Central Register of Controlled Trials, and Web of Science to identify relevant studies from inception to January 2018. There will be no limitations on the publication languages. The search process was designed using following keywords:

Peritoneoscope∗, Celioscope∗, Laparoscope∗, Laparoscopic, Laparoscopy, Positive end-expiratory pressure, PEEP, Positive pressure respiration, Positive pressure ventilation, random, and RCT. The reference lists of included trials and reviews identified from initial searches will be scanned for more relevant studies. Search strategy of PubMed was as follows:

#1 “Positive-Pressure Respiration”[Mesh]

#2 “Positive end-expiratory pressure”[Title/Abstract] OR “PEEP”[Title/Abstract] OR “Positive pressure respiration”[Title/Abstract] OR “Positive-Pressure Respirations”[Title/Abstract] OR “Respiration, Positive-Pressure”[Title/Abstract] OR “Respirations, Positive-Pressure”[Title/Abstract] OR “Positive-Pressure Ventilation”[Title/Abstract] OR “Positive Pressure Ventilation”[Title/Abstract] OR “Positive-Pressure Ventilations”[Title/Abstract] OR “Ventilation, Positive-Pressure”[Title/Abstract] OR “Ventilations, Positive-Pressure”[Title/Abstract] OR “End-Expiratory Pressure, Positive”[Title/Abstract] OR “End-Expiratory Pressures, Positive”[Title/Abstract] OR “Positive End Expiratory Pressure”[Title/Abstract] OR “Positive End-Expiratory Pressures”[Title/Abstract] OR “Pressure, Positive End-Expiratory”[Title/Abstract] OR “Pressures, Positive End-Expiratory”[Title/Abstract]

#3 #1 OR #2

#4 “Laparoscopy”[Mesh]

#5 “Laparoscop∗”[Title/Abstract] OR “Celioscop∗”[Title/Abstract] OR “Laparoscopic Assisted Surgery”[Title/Abstract] OR “Surgeries, Laparoscopic”[Title/Abstract] OR “Surgeries, Laparoscopic Assisted”[Title/Abstract] OR “Laparoscopic Surgery”[Title/Abstract] OR “Surgery, Laparoscopic Assisted”[Title/Abstract] OR “Peritoneoscop∗”[Title/Abstract] OR “Surgery, Laparoscopic”[Title/Abstract] OR “Laparoscopic Surgeries”[Title/Abstract] OR “Laparoscopic Assisted Surgeries”[Title/Abstract]

#6 #4 OR #5

#7 “Randomized Controlled Trials as Topic”[Mesh] OR “Randomized Controlled Trial” [Publication Type]

#8 “Randomized Controlled Trials”[Title/Abstract] OR “Randomized Controlled Trial”[Title/Abstract] OR “RCT”[Title/Abstract] OR “Clinical Trials, Randomized”[Title/Abstract] OR “Trials, Randomized Clinical”[Title/Abstract] OR “Controlled Clinical Trials, Randomized”[Title/Abstract] OR “randomly”[Title/Abstract]

#9 #7 OR #8

#10 #3 AND #6 AND #9

### 2.4 Study selection

We will obtain the titles and abstracts of relevant literature from the database search techniques outlined in the search strategies. Two reviewers will independently screen and categorize all related articles, and the full texts of any potentially eligible studies will be retrieved independently by the same reviewers. Multiple submissions or duplicate publications will be compared and the more detailed one will be retained. If the same population was used in multiple studies, the article with the longest follow-up will be included. Methodological experts will be consulted to reach consensus if necessary. The reasons for the exclusion of any articles will be recorded, and any disagreement between them over the eligibility of particular studies will be resolved through discussion with a third reviewer.

### 2.5 Data extraction

Two reviewers will independently extract the required data from the studies selected for inclusion using Microsoft Excel 2013 (Microsoft Corp, Redmond, WA, www.microsoft.com). Data will be extracted from eligible studies including publication details, general characteristics of included trials (name of first author, year of publication, number of center, setting, total sample size, and inclusion and exclusion criteria), details of participants (gender, age, body mass index, operation time, and type of surgery), and intervention characteristics as well as outcomes. Any missing data will be acquired by contacting the author by email or telephone.

### 2.6 Risk of bias assessment

Two authors will independently assess the methodological quality of the included studies using the Cochrane risk of bias assessment tool (Cochrane Handbook for Systematic Reviews of Interventions), which includes reference to the following items: random sequence generation, allocation concealment, blinding of participants and personnel, blinding of outcome assessment, incomplete outcome data, selective reporting, and other bias. Every parameter will be classified into 3 categories (low risk, high risk, and unclear risk). Any disagreement between the reviewers on the risk of bias will be resolved by discussion and, if necessary, by consulting a third reviewer.

### 2.7 Statistical analysis

An NMA will be conducted on both direct evidence and indirect evidence, with the benefit of randomization in each study retained and the NMA will be conducted in a Bayesian framework using a random-effects model. A consistency model will be drawn for each evaluated outcome and the relative effect size of the treatment will be calculated using the mean difference for the continuous variables. We will use the node splitting method to examine the inconsistency between direct and indirect comparisons if a loop connecting 3 or more arms exist.^[28]^ If node-splitting analysis determined *P* > 0.05, the consistency model will be used for pooled analysis. Otherwise, the inconsistency model will be used.^[29,30]^ Additionally, the convergence will be assessed using the potential scale reduction factor and the Brooks–Gelman–Rubin method, and a value of 1 indicates a good convergence.^[31]^ We will also rank each treatment according to the probability that one is superior to the other. If there are 10 or more studies in the NMA, we will use the funnel plot to evaluate the potential publication bias.

## 3 Discussion

Several studies have observed the good effects of PEEP applications in laparoscopic surgery, such as improved gas exchange, counteracted intraoperative atelectasis, improved respiratory mechanics, and ventilation-perfusion abnormalities. Although 5 to 15 cm H_2_O PEEP was indicated to be most effective through the method of lung pressure volume curve analysis, high-resolution computed tomography, and monitoring for dynamic compliance, only a small number of large-scale, high-quality RCTs provide direct comparison of different PEEP, the standard level of PEEP during laparoscopy remains unknown, due to the relative paucity of clinical evidence. The present systematic review, based on NMA, will identify all relevant evidence to compare the effect of different PEEP levels during laparoscopic surgeries on respiratory mechanics and oxygenation. And to the best of our knowledge, this is the first NMA to explore this field, allowing us to synthesize randomized evidence for multiple treatment comparisons involving interventions without direct pair-wise comparisons. The results of the NMA will be submitted to peer-reviewed journals for publication and will be sent to the primary clinicians and healthcare professionals in the National Health Service, which is expected to help clinicians make more accurate treatment decisions and promote the development of research on PEEP application during surgery.

## Author contributions

Min Wang, Nan Geng, and Bo Wang planned and designed the study. Ya Gao, Yan Zhang, Yingbin Wang, and Xiping Shen tested the feasibility of the study. Jinhui Tian and Bo Wang provided methodological advice, polished and revised the manuscript. Min Wang, Nan Geng, and Bo Wang wrote the manuscript. All authors approved the final version of the manuscript.

**Conceptualization:** Min Wang, Nan Geng, Bo Wang.

**Data curation:** Yan Zhang, Xiping Shen.

**Formal analysis:** Yingbin Wang, Xiping Shen.

**Investigation:** Min Wang, Nan Geng, Ya Gao, Yan Zhang.

**Methodology:** Jinhui Tian, Bo Wang.

**Project administration:** Bo Wang.

**Resources:** Min Wang, Nan Geng.

**Software:** Ya Gao, Xiping Shen.

**Supervision:** Jinhui Tian, Bo Wang.

**Validation:** Yingbin Wang.

**Visualization:** Ya Gao, Yan Zhang.

**Writing – original draft:** Min Wang, Nan Geng.

**Writing – review & editing:** Jinhui Tian, Bo Wang.
